# Changes in Restrictive Spirometric Pattern and Pulmonary Function Before and After the COVID-19 Pandemic Among Korean Adults: A Nationwide Cross-Sectional Study

**DOI:** 10.3390/jcm15176771

**Published:** 2026-08-31

**Authors:** Taner Akbulut, Vedat Çinar, Do-Youn Lee

**Affiliations:** 1 Faculty of Sport Science, Fırat University, Elazig 23119, Türkiye; takbulut@firat.edu.tr (T.A.); vcinar@firat.edu.tr (V.Ç.); 2 College of General Education, Kookmin University, Seoul 02707, Republic of Korea

**Keywords:** restrictive pulmonary dysfunction, pulmonary function, COVID-19, Korea national health and nutrition examination survey, spirometry, forced vital capacity, epidemiology

## Abstract

**Background/Objectives:** A restrictive spirometric pattern (RSP), characterized by reduced forced vital capacity (FVC) with a preserved FEV1/FVC ratio, has been associated with adverse health outcomes and metabolic disorders. This study investigated differences in the prevalence of RSP and pulmonary function between 2019 and 2024 among Korean adults. **Methods:** This cross-sectional study analyzed data from the 2019 and 2024 Korea National Health and Nutrition Examination Survey. A total of 4856 adults aged ≥40 years without chronic obstructive pulmonary disease were included. RSP was defined as FVC < 80% predicted among participants with an FEV1/FVC ratio ≥ 0.70. Pulmonary function and RSP prevalence were compared between survey years using complex sample analyses. Multivariable logistic regression was performed to examine the association between the post-pandemic period and RSP. **Results:** The prevalence of RSP increased significantly from 2.31% in 2019 to 3.58% in 2024 (*p* = 0.025). FVC, FEV1, and FVC percent predicted significantly decreased after the COVID-19 pandemic (all *p* < 0.001), whereas the FEV1/FVC ratio (*p* = 0.166) and peak expiratory flow (*p* = 0.671) remained unchanged. After adjustment for demographic characteristics, health behaviors, and metabolic factors, the post-pandemic period remained independently associated with RSP (odds ratio, 1.571; 95% confidence interval, 1.036–2.380; *p* = 0.033). **Conclusions:** The prevalence of RSP increased significantly after the COVID-19 pandemic and was accompanied by declines in lung volume-related pulmonary function without evidence of increased airflow obstruction. These findings highlight the importance of strategies targeting metabolic health, obesity, and physical function to preserve respiratory health in the post-pandemic era.

## 1. Introduction

Since the onset of the coronavirus disease 2019 (COVID-19) pandemic, substantial changes in daily routines, physical activity, and eating behavior have been reported across populations, with a general tendency toward less movement and greater sedentary time [1,2]. These shifts are clinically relevant because reduced activity, prolonged sedentary behavior, and unfavorable dietary patterns may promote weight gain, adverse metabolic changes, and reduced cardiopulmonary fitness [1,2]. Accordingly, the post-pandemic period warrants evaluation not only from a cardiometabolic perspective but also from a pulmonary health perspective.

A restrictive spirometric pattern (RSP), characterized by a reduced FVC with a preserved FEV1/FVC ratio, has been associated with increased morbidity and mortality and with metabolic abnormalities. However, spirometry alone cannot confirm a true restrictive ventilatory defect, which requires measurement of static lung volumes, particularly total lung capacity [3]. Previous studies have shown that restrictive spirometric impairment is associated with increased morbidity and mortality and is closely linked to systemic metabolic abnormalities, including metabolic syndrome and type 2 diabetes mellitus [3,4,5]. In particular, obesity and abdominal obesity may reduce lung volumes by limiting diaphragmatic excursion and chest wall compliance, while chronic low-grade inflammation and insulin resistance may further contribute to impaired pulmonary function [4,5,6]. Taken together, these observations suggest that RSP may reflect systemic metabolic dysfunction as well as diminished respiratory reserves.

Most pulmonary studies conducted after the COVID-19 pandemic have focused on respiratory sequelae among patients recovering from SARS-CoV-2 infection, frequently emphasizing diffusion impairment or restrictive abnormalities during clinical follow-up [7]. However, this patient-based literature does not adequately address whether the prevalence of restrictive spirometric patterns has changed at the population level across the pandemic period. This distinction is important because adiposity- and metabolism-related declines in FVC could emerge in the general population even without a parallel increase in airflow obstruction.

Therefore, the present study investigated differences in the prevalence of RSP and pulmonary function parameters between Korean adults surveyed in 2019 and 2024 using nationally representative data. We further examined whether survey year was associated with RSP after accounting for demographic, behavioral, and metabolic factors. We hypothesized that the prevalence of RSP would be higher and FVC-related spirometric indices would be lower in 2024 than in 2019.

## 2. Materials and Methods

### 2.1. Study Population and Data Source

This study used data from the Korea National Health and Nutrition Examination Survey (KNHANES), a nationally representative cross-sectional survey conducted by the Korea Disease Control and Prevention Agency (KDCA). KNHANES employs a stratified, multistage probability sampling design to obtain representative health and nutritional data from the noninstitutionalized Korean population. In the present study, data from the 2019 and 2024 KNHANES were analyzed to compare RSP prevalence before and after the COVID-19 pandemic.

**Figure 1:**
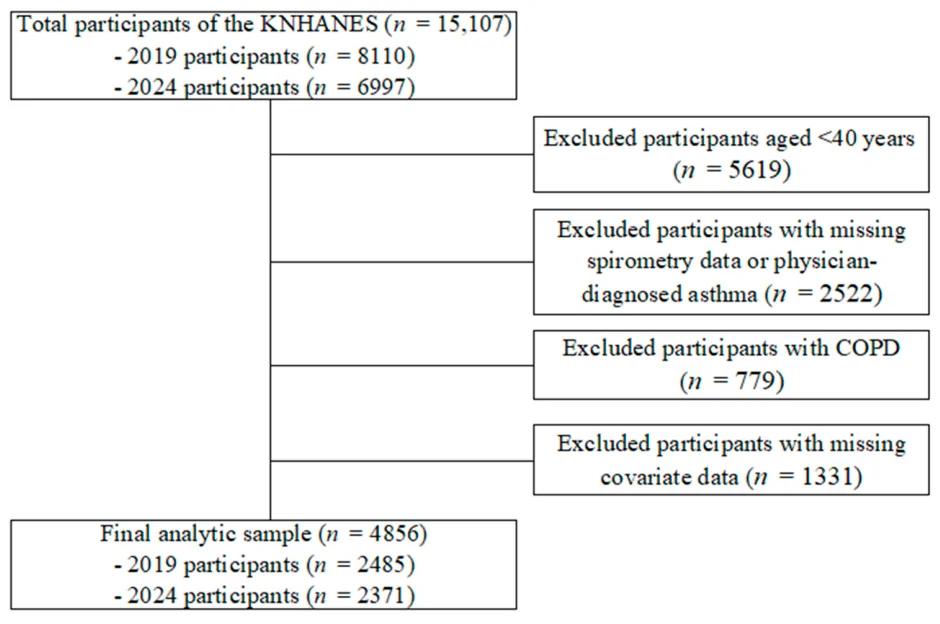
Flow chart of this study.

Among 15,107 participants from the 2019 and 2024 KNHANES, 5619 participants aged <40 years were excluded. Participants without valid spirometry data or with physician-diagnosed asthma were then excluded (*n* = 2522). Participants with chronic obstructive pulmonary disease, defined as an FEV1/FVC ratio < 0.70, were additionally excluded (n = 779). Finally, participants with missing data for major covariates were excluded (n = 1331). The final analytic sample consisted of 4856 participants, including 2485 participants from 2019 and 2371 participants from 2024. All analyses were conducted using complex sample weights, stratification variables, and primary sampling units provided by KNHANES (Figure 1).

### 2.2. Definition of Restrictive Pulmonary Dysfunction

Pulmonary function tests were performed according to standardized procedures using spirometry, consistent with internationally accepted ATS/ERS technical standards for spirometry [8,9]. Forced vital capacity (FVC), forced expiratory volume in 1 s (FEV1), FEV1/FVC ratio, and peak expiratory flow (PEF) were evaluated. An obstructive spirometric pattern was defined as an FEV1/FVC ratio < 0.70. RSP was operationally defined as an FEV1/FVC ratio ≥ 0.70 with FVC < 80% of the predicted value [10].

Because the 2024 KNHANES dataset did not provide directly calculated FVC percent predicted values, predicted FVC values were calculated using Korean reference equations based on sex, age, and height. For men, predicted FVC was calculated as 0.052 × height (cm) − 0.028 × age − 3.20, whereas for women, the equation was 0.041 × height (cm) − 0.018 × age − 2.69. FVC percent predicted was subsequently calculated as measured FVC divided by predicted FVC multiplied by 100 [11].

### 2.3. Sociodemographic and Health-Related Variables

Age was categorized into four groups: 40–49, 50–59, 60–69, and ≥70 years. Educational level was categorized as elementary school or lower, middle school, high school, and university or higher. Marital status was classified according to spouse cohabitation status (with or without spouse). Individual income was categorized into quartiles. Residential areas were classified as urban or rural.

Smoking status was categorized as current smoker, former smoker, or never smoker based on conventional cigarette and heated tobacco product use. Alcohol consumption was classified as current drinking or non-drinking status. Aerobic physical activity was defined according to KNHANES criteria based on moderate- or vigorous-intensity physical activity recommendations. Resistance exercise was defined as participation in muscle-strengthening exercise at least twice per week [12].

Body mass index (BMI) was categorized as low weight (<18.5 kg/m^2^), normal weight (18.5–22.9 kg/m^2^), overweight (23.0–24.9 kg/m^2^), or obesity (≥25.0 kg/m^2^). Hypertension was defined as systolic blood pressure ≥140 mmHg, diastolic blood pressure ≥90 mmHg, or current use of antihypertensive medication. Diabetes status was categorized as normal, prediabetes, or diabetes. Abdominal obesity was defined as waist circumference ≥90 cm in men and ≥85 cm in women [13].

### 2.4. Statistical Analysis

All statistical analyses were performed using SPSS version 27.0 (IBM Corp., Armonk, NY, USA). Because KNHANES uses a complex sampling design, analyses were conducted using stratification variables, cluster variables, and sample weights according to the KDCA analytical guidelines.

Continuous variables are presented as weighted means ± standard errors (SE), whereas categorical variables are presented as weighted percentages. Complex sample cross-tabulation analyses were used to compare categorical variables according to survey year and RSP status. Complex sample general linear models were used to compare pulmonary function parameters between the pre- and post-COVID-19 periods.

To evaluate the association between survey year and RSP prevalence, complex sample logistic regression analyses were performed. Odds ratios (ORs) and 95% confidence intervals (CIs) were calculated using the 2019 survey year as the reference group. Model 1 was adjusted for age, sex, educational level, marital status, individual income, and residential area. Model 2 was additionally adjusted for BMI, smoking status, alcohol consumption, aerobic physical activity, resistance exercise, hypertension, diabetes status, and abdominal obesity. A two-tailed *p*-value < 0.05 was considered statistically significant.

## 3. Results

### 3.1. General Characteristics of the Study Participants

Table 1 presents the general characteristics of the study participants before and after the COVID-19 pandemic. Significant differences in age distribution were observed between the two periods (*p* < 0.001). Compared with 2019, the proportion of participants aged 60 years and older increased in 2024, whereas the proportion of participants in their 40s decreased.

No significant differences were observed in sex distribution, marital status, personal income, region, smoking status, drinking status, or aerobic activity between the two periods. However, resistance exercise participation significantly increased after the COVID-19 pandemic (21.62% vs. 26.81%, *p* < 0.001). Regarding obesity-related factors, the prevalence of obesity increased significantly from 35.80% in 2019 to 39.37% in 2024 (*p* = 0.021). In metabolic health variables, the prevalence of prediabetes decreased, whereas the prevalence of diabetes increased significantly after the COVID-19 pandemic (*p* < 0.001). These findings suggest that substantial demographic and metabolic changes occurred following the COVID-19 pandemic.

### 3.2. Weighted Distribution of RSP According to Participant Characteristics

Table 2 shows the weighted distribution of RSP according to participant characteristics before and after the COVID-19 pandemic. In 2019, RSP was significantly associated with sex, education level, and BMI category. The proportion of men was significantly higher in the RSP group compared with the normal pulmonary function group (77.38% vs. 44.39%, *p* < 0.001). In addition, participants with lower educational attainment and obesity showed a higher prevalence of RSP.

In 2024, the associations became more pronounced across several variables. Older age groups demonstrated significantly higher proportions of RSP, particularly among participants aged 70 years and older (37.60%, *p* < 0.001). Men continued to show a significantly higher prevalence of RSP than women (66.84% vs. 44.16%, *p* < 0.001). Smoking status was also significantly associated with RSP after the COVID-19 pandemic, with current smokers accounting for a greater proportion in the RSP group (25.16%, *p* = 0.036).

Furthermore, obesity-related and metabolic factors demonstrated strong associations with RSP in 2024. Participants with obesity showed a markedly higher prevalence of RSP (55.45%, *p* = 0.003), while hypertension, diabetes, and abdominal obesity were also significantly more prevalent in the RSP group (all *p* < 0.01). These findings indicate differences in the distribution of obesity-related and metabolic factors according to RSP status in 2024.

### 3.3. Comparison of Pulmonary Function Parameters Before and After the COVID-19 Pandemic

Table 3 compares pulmonary function parameters between the pre- and post-COVID-19 periods. Forced vital capacity (FVC) significantly decreased from 3.45 ± 0.02 L in 2019 to 3.39 ± 0.03 L in 2024 (*p* < 0.001). Similarly, forced expiratory volume in one second (FEV1) decreased significantly after the COVID-19 pandemic (2.72 ± 0.02 L vs. 2.69 ± 0.02 L, *p* < 0.001).

The predicted forced vital capacity percentage (FVCp) also showed a significant decline after the COVID-19 pandemic (104.07 ± 0.31 vs. 102.83 ± 0.35, *p* < 0.001). In contrast, no significant difference was observed in the FEV1/FVC ratio between the two periods (*p* = 0.166). No significant difference was observed in peak expiratory flow (PEF) between 2019 and 2024 (*p* = 0.671). Overall, these findings indicate modest population-level differences in spirometric parameters between 2019 and 2024, characterized by lower FVC, FEV1, and FVC percent predicted in 2024, while the FEV1/FVC ratio and PEF remained unchanged.

### 3.4. Age-Specific Prevalence of RSP According to Survey Year

Table 4 presents the age-specific prevalence of RSP according to survey year. The overall prevalence of RSP increased significantly from 2.31% in 2019 to 3.58% in 2024 (*p* = 0.025). Although the prevalence of RSP slightly decreased among participants in their 40s, increasing trends were observed in participants aged 50 years and older. In particular, the prevalence of RSP among adults aged 70 years and older increased from 3.89% in 2019 to 7.65% in 2024, showing the largest absolute increase among all age groups, although the difference was not statistically significant (*p* = 0.058). Similarly, the prevalence of RSP increased in participants in their 50s and 60s; however, these differences were not statistically significant. Overall, the prevalence of RSP was significantly higher in 2024 than in 2019. Among participants who otherwise met the eligibility criteria, 49 participants in 2019 and 24 participants in 2024 had an obstructive spirometric pattern (FEV1/FVC < 0.70) with concomitantly reduced FVC (<80% predicted).

### 3.5. Odds Ratios for RSP in 2024 Compared to 2019

Table 5 presents the odds ratios (ORs) for RSP in 2024 compared with 2019. In the crude model, the odds of RSP were significantly higher in 2024 than in 2019 (OR = 1.571, 95% CI = 1.054–2.344, *p* = 0.027). After adjustment for demographic variables including age, sex, education, marital status, personal income, and region (Model 1), the association remained statistically significant (OR = 1.503, 95% CI = 1.005–2.249, *p* = 0.047). Further adjustment for health behavior and metabolic factors, including BMI, smoking, drinking, aerobic activity, resistance exercise, and metabolic health variables (Model 2), also showed significantly increased odds of RSP after the COVID-19 pandemic (OR = 1.571, 95% CI = 1.036–2.380, *p* = 0.033). These results indicate that the 2024 survey was associated with higher odds of RSP compared with 2019 after adjustment for demographic characteristics, health behaviors, and metabolic factors.

## 4. Discussion

The primary objective of this study was to evaluate population-level differences in the prevalence of RSP and spirometric parameters among Korean adults surveyed in 2019 and 2024 using nationally representative KNHANES data. The prevalence of RSP was significantly higher in 2024 than in 2019 (3.58% vs. 2.31%). FVC, FEV1, and FVC percent predicted were also modestly lower in 2024, whereas the FEV1/FVC ratio and PEF did not differ significantly between the two survey years. In multivariable analysis, the 2024 survey year remained associated with higher odds of RSP after adjustment for demographic characteristics, health behaviors, and metabolic factors. These findings demonstrate temporal differences in spirometric patterns at the population level but do not establish that the COVID-19 pandemic or pandemic-related exposures caused these differences.

Stratified analysis by age cohort revealed that the prevalence of RSP was higher among older adults in 2024, with the largest absolute rise occurring in the oldest demographic. Specifically, the prevalence of RSP among individuals aged 70 years and older nearly doubled, climbing from 3.89% in 2019 to 7.65% in 2024. Several physiological and behavioral factors may potentially contribute to this age-related pattern. Normal aging is inherently linked with structural modifications of the respiratory system—a clinical state often conceptualized as “presbypnea”—characterized by progressive calcification of the costal cartilages, increased stiffness of the chest wall, and a reduction in lung parenchyma elasticity [4]. This age-related reduction in physiological compliance is further compounded by “respiratory sarcopenia,” defined as the systemic loss of skeletal muscle mass and functional efficiency that directly weakens the diaphragm and accessory inspiratory muscles [5]. Since skeletal muscle mass is a major determinant of FVC and FEV1, age-associated muscle atrophy directly compromises ventilatory capacity. Pandemic-related reductions in physical activity and physical deconditioning have been reported in older populations and may represent possible explanations; however, these mechanisms could not be directly evaluated in the present study [6,7].

Furthermore, this study highlights a persistent and pronounced sex disparity, with men constituting the vast majority of the RSP population in both periods, comprising 77.38% of the RSP cohort in 2019 and 66.84% in 2024. This striking male-centric distribution is consistent with contemporary KNHANES epidemiologic analyses indicating that the metabolic and physical health profiles of Korean men deteriorated much more severely than those of women during the COVID-19 pandemic [14,15]. Under pandemic restrictions, the prevalence of obesity and metabolic syndrome rose dramatically among Korean adult men, whereas women maintained a relatively stable trend [16,17]. From an anatomical and anthropometric perspective, men are significantly more susceptible to the accumulation of visceral adipose tissue within the intra-abdominal and mediastinal cavities compared to women in similar BMI categories, which exerts greater passive mechanical compression on the thoracic cage [1]. Furthermore, current smoking emerged as a significant modifier in 2024, with active smokers comprising 25.16% of the RSP cohort compared to only 14.98% of the normal group (*p* = 0.036). These factors may contribute to reduced FVC in some individuals; however, the present study cannot determine whether reduced FVC reflects air trapping, true restriction, or other physiological mechanisms.

The observed association between RSP and adiposity-related markers suggests that obesity may be one of several factors associated with reduced vital capacity at the population level. In KNHANES 2024, the proportion of participants with general obesity (BMI ≥ 25.0 kg/m^2^) significantly increased to 39.37% from 35.80% in 2019, and general obesity was present in 55.45% of the RSP group compared to only 38.77% of the normal lung function group (*p* = 0.003). More critically, abdominal obesity, defined by waist circumference, exhibited an even stronger association with RSP, affecting 63.80% of the RSP cohort in 2024 compared to 37.21% of the normal cohort (*p* < 0.001), which represents a notable increase from the non-significant association observed in 2019 (*p* = 0.250). The pathophysiology of obesity-induced restriction centers on the mechanical mass-loading of adipose tissue over the chest wall and within the visceral abdominal cavity [18]. The physical weight of anterior chest wall fat and the pressure exerted by intra-abdominal visceral adipose tissue displace the diaphragm cephalad into the thoracic cavity and limit its downward excursion during inspiration [19]. Consequently, there is an exponential reduction in respiratory system compliance and dynamic lung compliance, causing individuals to breathe at abnormally low operating volumes [20]. This mechanical compression decreases functional residual capacity (FRC) and expiratory reserve volume (ERV) to levels near or below the closing volume of the lung, which triggers premature airway closure in dependent lung zones, microatelectasis, and regional ventilation–perfusion mismatching.

Importantly, the clinical manifestations of RSP cannot be attributed entirely to mechanical restrictive forces; rather, they reflect a wider state of systemic metabolic dysfunction and chronic low-grade inflammation [21]. In the KNHANES 2024 cohort of this study, RSP was heavily clustered with major cardiometabolic comorbidities, with affected individuals exhibiting significantly higher rates of hypertension (55.72% vs. 38.95%, *p* = 0.005) and type 2 diabetes mellitus (41.26% vs. 17.68%, *p* < 0.001) compared to the normal population. Visceral adipose tissue operates as a highly active endocrine organ that recruits macrophages and chronically secretes pro-inflammatory adipokines, including tumor necrosis factor-alpha (TNF-alpha), interleukin-6 (IL-6), and C-reactive protein (CRP), into the systemic circulation. This persistent inflammatory state directly impairs insulin signaling pathways, driving systemic insulin resistance and progressive hyperglycemia. Chronic exposure to high glucose levels and systemic inflammatory mediators induces microangiopathy within the extensive pulmonary capillary network, characterized by thickening of the alveolar-capillary basement membrane, endothelial cell dysfunction, and interstitial fibrotic remodeling [22]. These structural alterations reduce lung tissue compliance and compromise gas exchange efficiency, manifesting spirometrically as a restricted vital capacity [23]. These mechanisms may partly explain previously reported associations between metabolic abnormalities and reduced lung volumes. However, the cross-sectional design of the present study does not permit determination of the direction or underlying mechanisms of these associations.

The changes in health behaviors observed during the post-pandemic period also reveal a critical counterbalancing factor: while aerobic physical activity rates remained statistically unchanged (41.05% in 2019 vs. 41.70% in 2024), participation in resistance exercise significantly increased from 21.62% to 26.81% (*p* < 0.001). This rise in muscle-strengthening exercise suggests that a portion of the population adopted home-based resistance training to maintain physical fitness amid pandemic restrictions. Resistance training plays a pivotal role in preserving respiratory health through direct and indirect pathways. Unlike aerobic exercises, strength training directly enhances the structural capacity and endurance of the respiratory musculature, including the diaphragm and intercostal muscles, which are essential for maximizing chest wall expansion and maintaining vital capacity. Moreover, resistance training has been shown to be highly effective in suppressing visceral fat accumulation and central adiposity, independent of caloric restriction. KNHANES-based studies have demonstrated that performing strength training at least once a week significantly suppresses abdominal obesity, with odds ratios of 0.634 in 2019 and 0.614 in 2020, even after adjusting for total energy intake and age. Resistance exercise may contribute to reductions in visceral adiposity and preservation of skeletal muscle mass, which could potentially reduce mechanical loading on the thorax and systemic inflammation [24]. These findings from previous studies suggest potential pathways through which resistance exercise may be associated with respiratory and metabolic health.

The higher prevalence of RSP observed in 2024 may warrant further investigation, particularly because restrictive spirometric patterns have been associated with adverse health outcomes in previous studies [25]. It should also be emphasized that the absolute differences in pulmonary function parameters were small. FVC percent predicted remained above 100% in both survey years, and the mean absolute difference in FVC was approximately 60 mL. Therefore, although these differences were statistically significant at the population level, their clinical significance at the individual level should be interpreted cautiously. Clinicians frequently dismiss a reduced FVC with a normal FEV1/FVC ratio as a clinically insignificant or false-positive finding, prioritizing obstructive pathologies such as asthma and COPD. However, patients presenting with a restrictive spirometric pattern, even when classified as “mild” based on an FVCp between 50% and 80%, experience a substantial burden of chronic respiratory symptoms, including exertional dyspnea, sputum production, and wheezing. Validated quality-of-life assessments, such as the St. George’s Respiratory Questionnaire (SGRQ), demonstrate that individuals with RSP score similarly to those with established COPD, underscoring that their functional impairment is comparable in daily life. Furthermore, RSP is associated with a nearly two-fold increase in all-cause mortality, cardiovascular mortality, and respiratory-related mortality, independent of baseline BMI or smoking history [17]. Recognizing RSP as a systemic cardiorespiratory risk marker rather than an isolated spirometric variation is essential for early clinical screening, risk stratification, and the targeted prevention of cardiometabolic multimorbidity in vulnerable populations.

This study has several limitations. First, the repeated cross-sectional design of KNHANES precludes assessment of individual longitudinal changes and causal inference regarding the observed differences between 2019 and 2024. Second, static lung volumes, including total lung capacity (TLC), were unavailable; therefore, the spirometric definition used in this study represents an RSP rather than a confirmed restrictive ventilatory defect, and true restriction could not be distinguished from pseudorestriction. Participants with an obstructive spirometric pattern and concomitantly reduced FVC were classified as obstructive and excluded from the RSP analysis, and the underlying mechanism of reduced FVC in this subgroup could not be determined. Third, post-bronchodilator spirometry was unavailable, and reversible airflow limitation could not be completely excluded. Information on previous SARS-CoV-2 infection, disease severity, hospitalization, post-COVID syndrome, chest imaging, and DLCO was also unavailable; therefore, the observed differences cannot be attributed to individual COVID-19 infection or post-COVID pulmonary sequelae. Fourth, the use of fixed spirometric thresholds and Korean reference equations rather than GLI-based LLN or z-scores may have introduced age-related misclassification and may limit comparability with studies using contemporary international standards. Despite these limitations, the use of a large, nationally representative sample and a complex sampling design support the generalizability of the findings to the Korean adult population.

## 5. Conclusions

In conclusion, the prevalence of RSP was significantly higher among Korean adults surveyed in 2024 than among those surveyed in 2019. FVC, FEV1, and FVC percent predicted were modestly lower in 2024, whereas the FEV1/FVC ratio and PEF remained unchanged. The 2024 survey year was also associated with higher odds of RSP after adjustment for demographic characteristics, health behaviors, and metabolic factors. These findings demonstrate population-level temporal differences in spirometric patterns between 2019 and 2024 but do not establish a causal effect of the COVID-19 pandemic. Given the small absolute differences in pulmonary function and the absence of static lung volume measurements, the clinical significance and underlying mechanisms of these findings should be interpreted cautiously. Further longitudinal studies incorporating comprehensive pulmonary function assessments are warranted.

## Figures and Tables

**Table 1:** General characteristics of the study participants according to survey year.

Factors	Categories	Before (Weighted %)	After (Weighted %)	*p*
Age	40s	32.17	25.91	<0.001
	50s	32.43	29.5	
	60s	21.15	26.97	
	70s+	14.25	17.61	
Sex	Men	45.15	44.97	0.9
	Women	54.85	55.03	
Education	Elementary	13.52	11.19	0.097
	Middle	11.59	11.9	
	High	34.17	31.56	
	University	40.71	45.4	
Spouse	With	87.4	85.81	0.209
	Without	12.6	14.19	
Personal income	Q1 (Lowest)	21.43	19.52	0.554
	Q2	24.69	26.72	
	Q3	26.71	25.9	
	Q4 (Highest)	27.17	27.9	
Region	Urban	82.9	83.12	0.956
	Rural	17.1	16.88	
Smoking status	Current	16.53	15.34	0.518
	Former	24.26	23.64	
	Never	59.21	61.0	
Drinking status	Current	52.36	54.08	0.326
	Non-Drinker	47.64	45.92	
Aerobic activity	Yes	41.05	41.70	0.708
Resistance exercise	Yes	21.62	26.81	<0.001
BMI	Underweight	1.91	2.73	0.021
	Normal	35.5	33.9	
	Overweight	26.8	24	
	Obesity	35.8	39.37	
Metabolic health	Hypertension	36.31	39.55	0.066
	Pre-/Diabetes	49.21/15.86	38.06/18.53	<0.001
	Abdominal obesity	37.56	38.17	0.716

**Table 2:** Weighted distribution of RSP according to participant characteristics and survey year.

		Before (2019)			After (2024)		
Factors	Categories	Normal	RSP	*p*	Normal	RSP	*p*
Age	40s	32.31	26.09	0.311	26.4	12	<0.001
	50s	32.51	29.11		29.7	25.5	
	60s	21.16	20.82		27	24.9	
	70s+	14.02	23.98		16.9	37.6	
Sex	Men	44.39	77.38	<0.001	44.16	66.84	<0.001
	Women	55.61	22.62		55.84	33.16	
Education	Elementary	13.48	15.17	0.025	11.13	12.8	0.072
	Middle	11.23	26.85		11.5	21.53	
	High	34.38	25.32		31.67	28.45	
	University	40.9	32.66		45.7	37.23	
Spouse	With	87.28	92.4	0.176	85.69	88.97	0.399
	Without	12.72	7.6		14.31	11.03	
Personal income	Q1 (Lowest)	21.45	20.8	0.298	19.78	12.37	0.235
	Q2	24.45	34.66		26.4	35.28	
	Q3	26.71	26.74		25.85	26.45	
	Q4 (Highest)	27.39	17.8		27.97	25.89	
Region	Urban	82.87	84.25	0.767	83.09	83.9	0.859
	Rural	17.13	15.75		16.91	16.1	
Smoking status	Current	16.5	17.57	0.071	14.98	25.16	0.036
	Former	23.94	37.76		23.5	27.53	
	Never	59.55	44.66		61.52	47.3	
Drinking status	Current	52.28	55.72	0.616	54.37	46.21	0.193
	Non-Drinker	47.72	44.28		45.63	53.79	
Aerobic activity	Yes	41.00	43.39	0.741	41.81	38.87	0.611
Resistance exercise	Yes	21.60	22.25	0.92	26.79	27.48	0.902
BMI	Underweight	1.81	6.65	0.012	2.6	6.33	0.003
	Normal	35.97	16.36		34.45	19.03	
	Overweight	26.6	34.28		24.18	19.19	
	Obesity	35.63	43.01		38.77	55.45	
Metabolic health	Hypertension	35.99	49.59	0.075	38.95	55.72	0.005
	Pre-/Diabetes	49.02/15.70	57.43/22.60	0.088	37.88/17.68	42.69/41.26	<0.001
	Abdominal obesity	37.35	46.46	0.25	37.21	63.8	<0.001

**Table 3:** Comparison of pulmonary function parameters and prevalence of RSP before and after the COVID-19 pandemic.

Factors	Categories	COVID-19		*p*
		Before (M ± SE)	After (M ± SE)	
Pulmonary function	FVC	3.45 ± 0.02	3.39 ± 0.03	<0.001
	FEV1	2.72 ± 0.02	2.69 ± 0.02	<0.001
	FEV1/FVC	0.79 ± 0.00	0.80 ± 0.00	0.166
	FVCp	104.07 ± 0.31	102.83 ± 0.35	<0.001
	PEF	7.08 ± 0.05	7.11 ± 0.06	0.671

**Table 4:** Age-specific prevalence of RSP according to survey year.

Age Group	2019 Prevalence	2024 Prevalence	*p*
40s	1.87	1.67	0.805
50s	2.07	3.09	0.297
60s	2.27	3.31	0.313
≥70	3.89	7.65	0.058
Total	2.31	3.58	0.025

**Table 5:** Odds ratios for RSP in 2024 compared to 2019.

	Model	OR (95% CI)	*p*
RSP	Crude	1.571 (1.054–2.344)	0.027
	Model 1	1.503 (1.005–2.249)	0.047
	Model 2	1.571 (1.036–2.380)	0.033

Reference category: Before COVID-19 (2019). Model 1: age, sex, education, marital status, individual income, region. Model 2: Model 1 + BMI, smoking, drinking, aerobic, resistance exercise, metabolic health.

## Data Availability

All data were anonymized and can be downloaded from the website (https://knhanes.kdca.go.kr/knhanes, accessed on 8 June 2026).
